# Supplementary material for: Intake of Natural, Unprocessed Tiger Nuts (Cyperus esculentus L.) Drink Significantly Favors Intestinal Beneficial Bacteria in a Short Period of Time
Source: Nutrients. 2022 Apr 20;14(9):1709. doi: 10.3390/nu14091709 (PMC9104503; doi:10.3390/nu14091709)
Supplement: Supplementary file 1 [file nutrients-14-01709-s001.zip › nutrients-1673022-supplementary.pdf]

Supplementary Materials

Figure S1. Global microbiota distribution in samples and overrepresented genera among the five clusters identified in the text and in Figure 1. (**A**) Beta diversity Principal coordinates analysis (PCoA) derived from Bray Curtis distances among samples (p = 0 003 by PERMANOVA). (**B**) Microbial clusters and representative microbial taxa at genus level according to migration groups between baseline clusters and after the intervention. (**C**) LEfSe analysis indicates differentially abundant genus in each cluster.

A)

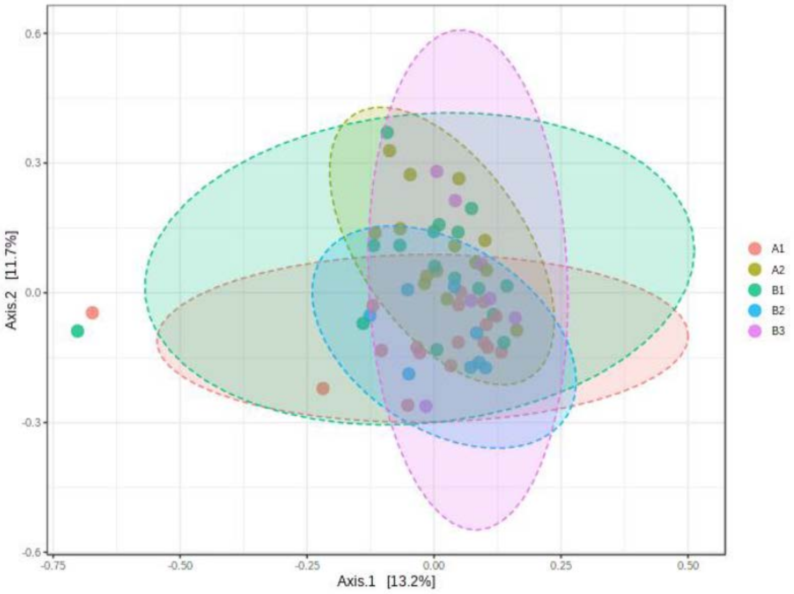

B)

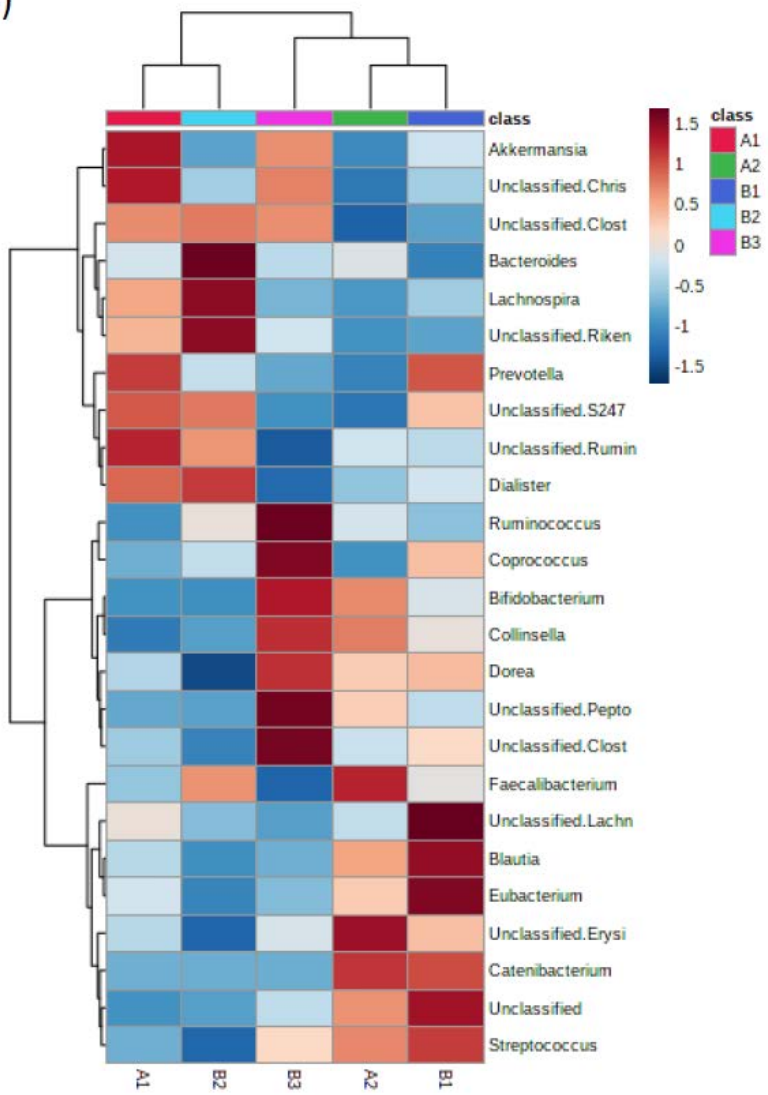

class
Akkermansia
Unclassified.Chris
Unclassified.Clost
Bacteroides
Lachnospira
Unclassified.Riken
Prevotella
Unclassified.S247
Unclassified.Rumin
Dialister
Ruminococcus
Coprococcus
Bifidobacterium
Collinsella
Dorea
Unclassified.Pepto
Unclassified.Clost
Faecalibacterium
Unclassified.Lachn
Blautia
Eubacterium
Unclassified.Erysi
Catenibacterium
Unclassified
Streptococcus
A1
B2
B3
A2
B1
1.5
1
0.5
0
-0.5
-1
-1.5
class
A1
A2
B1
B2
B3

C)

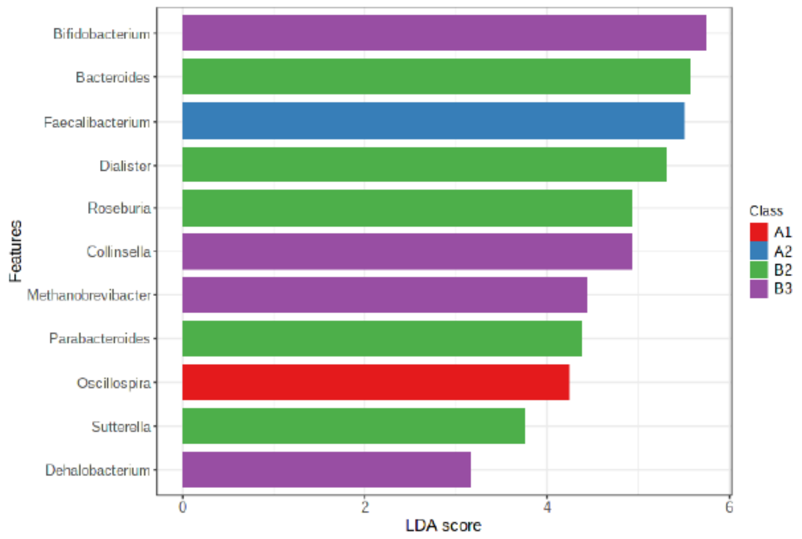

Bifidobacterium
Bacteroides
Faecalibacterium
Dialister
Roseburia
Collinsella
Methanobrevibacter
Parabacteroides
Oscillospira
Sutterella
Dehalobacterium
Features
0
2
4
6
LDA score
Class
A1
A2
B2
B3

Table S1. Validation of a FFQ by use of a 3-day recall.

	FFQ[1]	3 day-records[2]	R	*p*-value
Total protein	102.7±13.4	104.6±21.6	0.332	0.268
Animal protein	65.6±13.9	67.6±28.6	0.373	0.209
Plant protein	37.1±7.4	37.1±9.7	0.505	0.078
Lipids	111.3±13.4	114.9±15.5	0.710	0.007
Carbohydrates	262.7±35.7	261.2±49.7	0.668	0.013
Total dietary fiber	29.6±7.9	30.3±9.2	0.760	0.003

[1]Data presented as the mean ± standard deviation. Comparison of the nutrient intake using a 3-day diet records and the food frequency questionnaire.
[2] Association between nutrient intakes estimated by a food frequency questionnaire (FFQ) and 3-day diet records by use of Pearson correlation coefficient (R) test. P<0.05 was statistically significant.

Table S2. Nutrient composition and calories of freshly prepared natural horchata without added sugars supplied in this assay. The composition of standard natural horchata with added sugars is described, as well as that of different brands of bottled pasteurized commercial horchata where starch has been hydrolysed.

		Natural Horchata without added sugar*	Standard Natural Horchata	Bottled pasteurized Brand H†	Bottled pasteurized Brand P†	Bottled pasteurized Brand CU†	Bottled pasteurized Brand CM†
		**g/100 ml**	**g/100 ml**	**g/100 ml**	**g/100 ml**	**g/100 ml**	**g/100 ml**
Lipids		4.1	4.1	3.6	3.6	2,8	3,8
Total Carbohydrates		6.8	13.8	12	12	11	13
from which	Starch/ Fibre	5.5	5.5	1‡	1‡	1‡	1‡
	Simple sugars	1.3	6.1	11	11	10	12
Protein		1.2	1.2	1	1	0,6	0,9
kcal/100 ml		68.9	165.9	96.4	96.4	79.6	89.8

* supplied in this study.
† nutrients composition as described in the label of different commercial products.
‡ the estimated content of residual carbohydrate polymers of 1%, which is possibly due to the reported use of stabilizers described in the ingredients list.

Table S3. Proportions of predominant genera in the different Microbiota profiles before ad after the intervention.

		Cluster A1		Cluster A2	
		**Before Intervention**	**After Intervention**	**Before Intervention**	**After Intervention**
Cluster B1	*Akkermansia*	1.61 (3.86)	1.365 (7.19)	0.085 (0.44)	0.02 (0.18)
	Bacteroides	1.5 (1.27)	1.875 (1.49)	2.05 (1.57)	2.985 (3.86)
	Bifidobacterium	1.635 (2.69)	1.395 (1.99)	7.195 (5.09)	4.425 (7.23)
	Blautia	8.5 (11.24)	7.655 (3.57)	14.49 (4.50)	12.65 (9.30)
	Collinsella	1.24 (1.04)	0.735 (0.67)	1.36 (1.07)	2.11 (2.15)
	Coprococcus	5.27 (1.15)	3.28 (1.68)	6.515 (1.60)	4.525 (0.62)
	Dialister	0.01 (2.62)	0 (5.35)	0 (0.44)	0.015 (0.23)
	Dorea	2.12 (1.51)	1.725 (0.70)	1.97 (1.04)	2.255 (0.89)
	Eubacterium	2.775 (1.69)	2.335 (1.30)	0.045 (0.22)	0.06 (0.21)
	Faecalibacterium	3.23 (1.17)	4.61 (4.61)	6.65 (3.45)	6.87 (5.46)
	Lachnospira	0.145 (0.32)	0.075 (0.63)	0.33 (0.20)	0.14 (0.4)
	Oscillospira	0.77 (0.25)	0.9 (0.2)	0.545 (0.36)	0.73 (0.29)
	Prevotella	0.435 (1.19)	0.745 (1.71)	0 (0.04)	0.03 (0.10)
	Roseburia	0.295 (0.61)	0.315 (0.66)	0.705 (0.42)	0.255 (0.67)
	Ruminococcus	4.77 (2.64)	4.04 (1.59)	5.505 (0.74)	4.745 (1.90)

	Streptococcus	1.84 (3.55)	0.425 (0.76)	1.115 (0.68)	1.385 (2.52)
	Unclassified	1.375 (1.07)	1.085 (1.43)	3.48 (4.16)	2.865 (2.85)
	Un_Christensenellaceae	0.25 (0.8)	0.905 (1.47)	0.02 (0.08)	0.025 (0.03)
	Un.Clostridiaceae	0.375 (1.05)	0.73 (0.81)	1.15 (0.89)	0.435 (0.52)
	Un.Clostridiales	9.185 (7.61)	9.2 (5.39)	3.08 (1.53)	2.52 (3.39)
	Un.Coriobacteriaceae	0.565 (0.23)	0.395 (0.41)	0.58 (0.64)	0.575 (0.32)
	Un.Enterobacteriaceae	0.015 (0.12)	0.29 (1.11)	0.01 (0.01)	0.665 (1.40)
	Un.Erysipelotrichaceae	0.74 (0.73)	0.535 (0.59)	1.62 (1.93)	1.57 (2.76)
	Un.Lachnospiraceae	8.355 (4.39)	7.49 (3.78)	8.775 (7.56)	5.325 (6.6)
	Un.Peptostreptococcaceae	0.25 (0.22)	0.435 (0.26)	0.94 (1.08)	0.625 (1.01)
	Un.Ruminococcaceae	23.7 (13.21)	25.98 (7.58)	20.055 (8.96)	23.37 (11.78)
	Akkermansia	1.47 (3.44)	1.245 (4.16)	0.055 (0.05)	0.35 (0.35)
	Bacteroides	5.405 (5.9)	4.71 (1.48)	18.925 (5.81)	8.455 (5.04)
	Bifidobacterium	0.915 (1.66)	0.605 (1.17)	1.645 (1.39)	3.805 (2.80)
	Blautia	6.725 (4.8)	8.42 (3.13)	10.465 (4.00)	10.93 (1.9)
	Collinsella	0.695 (0.71)	0.725 (0.34)	1.12 (0.45)	1.23 (0.24)
	Coprococcus	5.335 (1.71)	6.65 (3.16)	3.345 (0.58)	5.16 (1.4)
	Dialister	3.07 (2.87)	3.4 (3.14)	4.405 (1.63)	5.085 (0.98)
	Dorea	1.415 (0.68)	1.515 (0.50)	2.025 (0.15)	1.33 (0.01)
	Eubacterium	0 (0)	0 (0)	2.13 (1.12)	4.085 (2.94)
	Faecalibacterium	6.74 (2.66)	4.515 (1.81)	9.11 (5.78)	13.27 (9.26)
	Lachnospira	0.66 (1.46)	0.69 (0.24)	0.52 (0.52)	0.04 (0.04)
	Oscillospira	0.77 (0.39)	0.93 (0.57)	0.68 (0.21)	0.425 (0.14)
	Prevotella	0.11 (1.46)	0.145 (0.58)	0.005 (0.01)	0.005 (0.01)
**Cluster B2**	*Roseburia*	1.265 (0.88)	0.66 (0.40)	3.01 (1.82)	1.755 (1.03)
	Ruminococcus	6.07 (1.19)	5.23 (1.91)	4.49 (0.46)	4.605 (1.83)
	Streptococcus	0.195 (0.38)	0.43 (0.79)	0.665 (0.65)	0.16 (0.11)
	Unclassified	1.3 (0.17)	1.295 (0.70)	2.285 (0.87)	2.365 (1.43)
	Un.Christensenellaceae	0.245 (0.31)	0.255 (0.67)	0.045 (0.02)	0.04 (0.03)
	Un.Clostridiaceae	0.51 (0.56)	0.36 (0.52)	0.72 (0.52)	0.4 (0.36)
	Un.Clostridiales	14.835 (4.52)	14.095 (4.76)	4.005 (2.58)	3.835 (2.74)
	Un.Coriobacteriaceae	0.27 (0.23)	0.265 (0.08)	0.22 (0.21)	0.275 (0.27)
	Un.Enterobacteriaceae	0.015 (0.17)	0.165 (1.08)	0.255 (0.26)	1.025 (0.61)
	Un.Erysipelotrichaceae	0.78 (0.51)	0.945 (0.79)	0.815 (0.04)	1.35 (0.89)
	Un.Lachnospiraceae	5.43 (1.89)	6.795 (3.5)	3.915 (0.30)	6.16 (3.95)
	Un Peptostreptococcaceae	0.265 (0.22)	0.45 (0.21)	0.895 (0.48)	0.855 (0.78)
	Un.Ruminococcaceae	26.295 (3.21)	26.38 (3.63)	17.035 (5.85)	20.88 (6.3)
	Akkermansia	1.75 (3.89)	8.68 (5.78)	0.325 (3.84)	0.38 (2.99)
	Bacteroides	6.07 (4.61)	5.6 (4.80)	1.3 (2.70)	1.895 (2.50)
	Bifidobacterium	3.46 (1.60)	0.83 (0.52)	17.555 (16.25)	9.62 (9.94)
	Blautia	6.66 (5.84)	3.27 (2.34)	8.43 (4.72)	5.755 (1.38)
	Collinsella	1.42 (1.48)	0.32 (0.71)	2.19 (2.51)	1.595 (1.45)
	Coprococcus	5.8 (1.29)	3.74 (1.94)	5.845 (2.11)	4.12 (2.19)
	Dialister	0 (0)	0 (0)	0.01 (0.21)	0.02 (0.83)
	Dorea	2.44 (0.36)	1.8 (0.13)	2.28 (0.63)	1.84 (0.63)
	Eubacterium	0.03 (0.06)	0.04 (0.02)	0.08 (1.8)	0.065 (1.57)
**Cluster B3**	*Faecalibacterium*	1.86 (1.02)	4.52 (1.82)	2.18 (2.75)	9.12 (4.99)
	Lachnospira	0.46 (0.13)	0.99 (0.45)	0.235 (0.15)	0.155 (0.22)
	Oscillospira	1.32 (0.40)	0.94 (0.2)	0.75 (0.59)	0.515 (0.17)
	Prevotella	0.49 (0.08)	0.32 (0.73)	0.015 (0.29)	0.035 (0.66)
	Roseburia	0.12 (0.07)	0.23 (0.09)	0.34 (0.33)	0.85 (0.65)
	Ruminococcus	9.09 (1.47)	6.96 (2.60)	6.605 (2.38)	7.695 (4.24)
	Streptococcus	0.35 (1.16)	0.63 (0.6)	1.5 (1.26)	0.82 (1.30)
	Unclassified	1.97 (0.61)	1.31 (0.19)	2.05 (0.87)	2.21 (1.22)
	Un.Christensenellaceae	2.77 (1.58)	2.35 (1.74)	0.14 (0.22)	0.175 (0.05)
	Un.Clostridiaceae	1.49 (0.44)	0.95 (0.42)	1.39 (1.09)	1.265 (0.45)

Un.Clostridiales	9.3 (2.25)	9.45 (0.92)	12.075 (6.32)	8.175 (7.5)
Un.Coriobacteriaceae	1.25 (0.34)	0.85 (0.99)	0.935 (1.16)	0.465 (0.42)
Un.Enterobacteriaceae	0.07 (0.02)	0.19 (0.24)	0.03 (0.07)	0.375 (0.86)
Un.Erysipelotrichaceae	1.16 (0.63)	2.69 (0.92)	1.415 (0.87)	1.305 (0.20)
Un.Lachnospiraceae	5.75 (3.54)	4.43 (1.22)	4.47 (2.45)	4.47 (2.14)
Un.Peptostreptococcaceae	0.68 (0.47)	0.46 (0.51)	1.425 (0.92)	0.555 (0.76)
Un.Ruminococcaceae	26.06 (2.05)	28.09 (1.02)	11.295 (6.18)	18.195 (7.00)

Data is expressed as median (interquartile range). Only those genera that had an averaged relative abundance higher than 0.5% were included in the table.
